# Supplementary figures and images for: Long-term outcomes following drug-coated balloons versus thin-strut drug-eluting stents for treatment of in-stent restenosis in Chronic Kidney Disease (CKD Dragon-Registry)
Source: PLoS One. 2025 Dec 29;20(12):e0337991. doi: 10.1371/journal.pone.0337991 (PMC12747340; doi:10.1371/journal.pone.0337991)

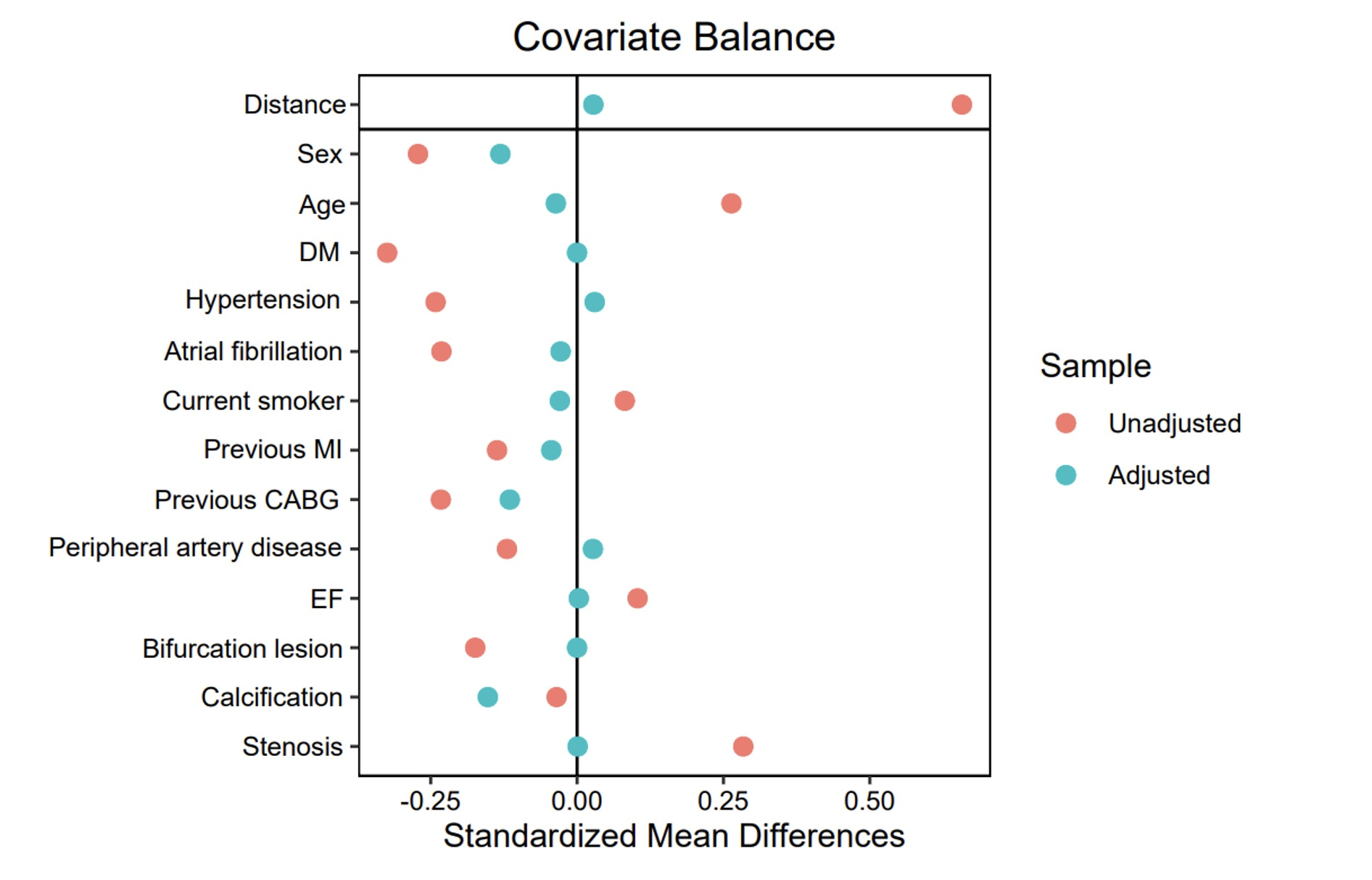

Supplement: S1 Fig — (JPG) [file pone.0337991.s001.jpg]

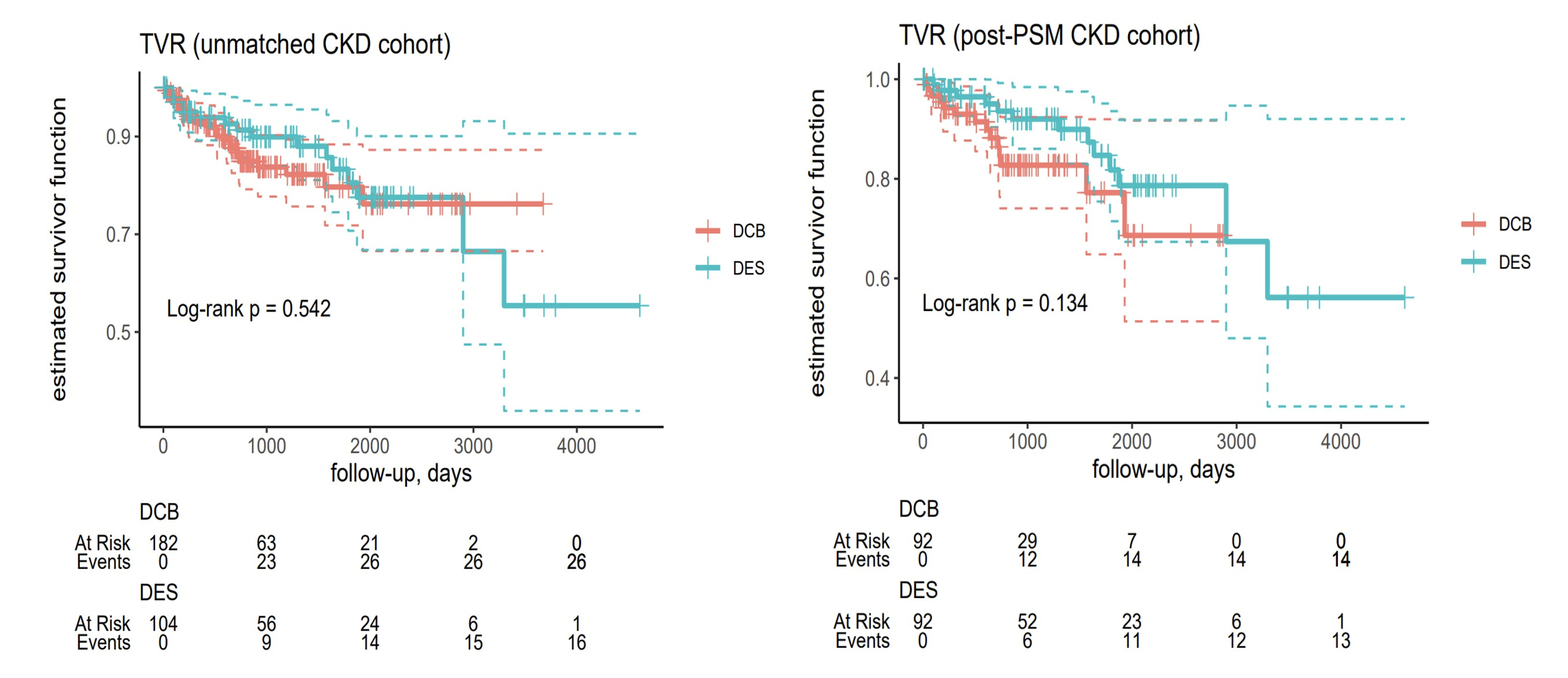

Supplement: S2 Fig — (JPG) [file pone.0337991.s002.jpg]

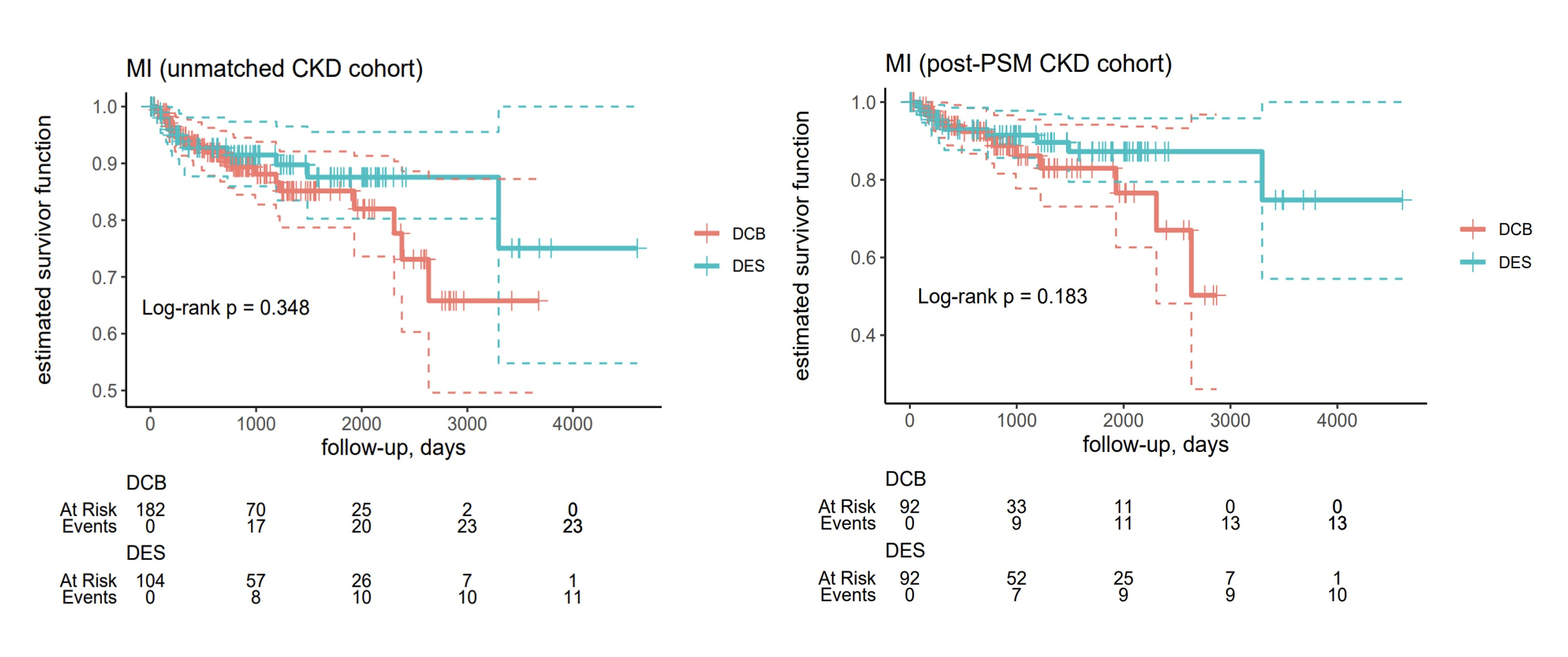

Supplement: S3 Fig — (JPG) [file pone.0337991.s003.jpg]

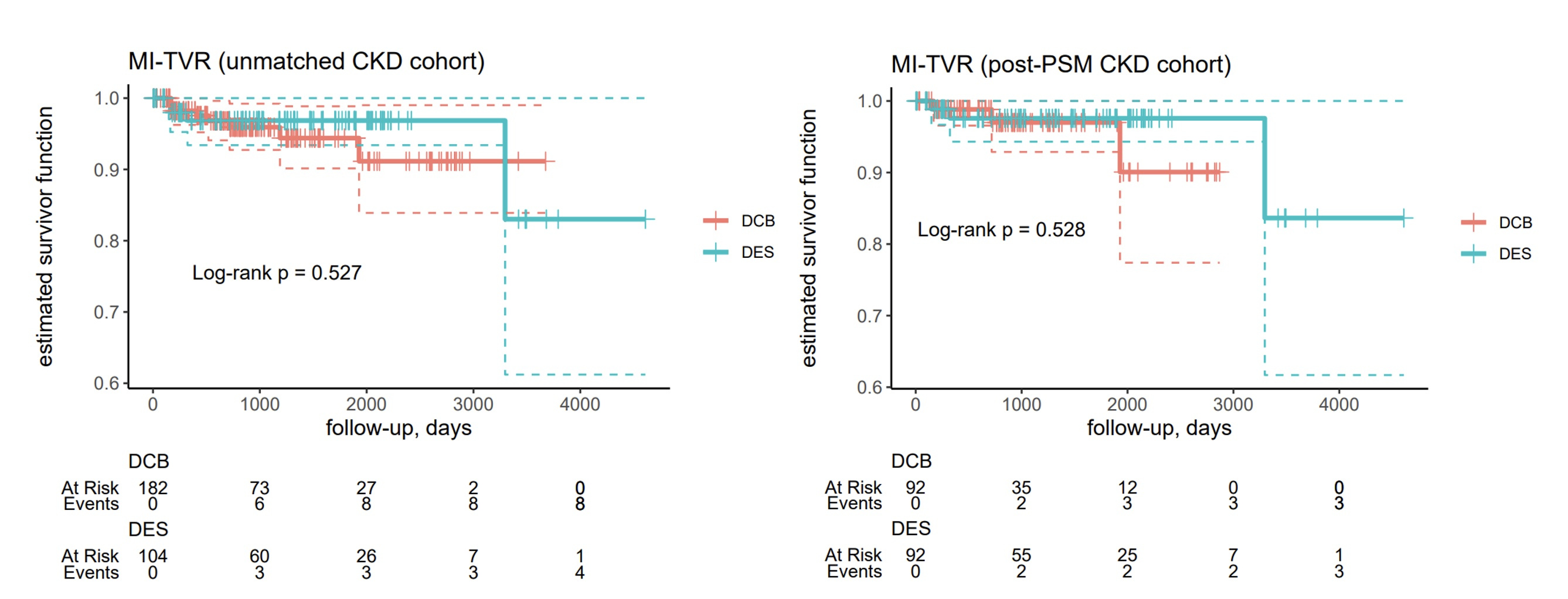

Supplement: S4 Fig — (JPG) [file pone.0337991.s004.jpg]

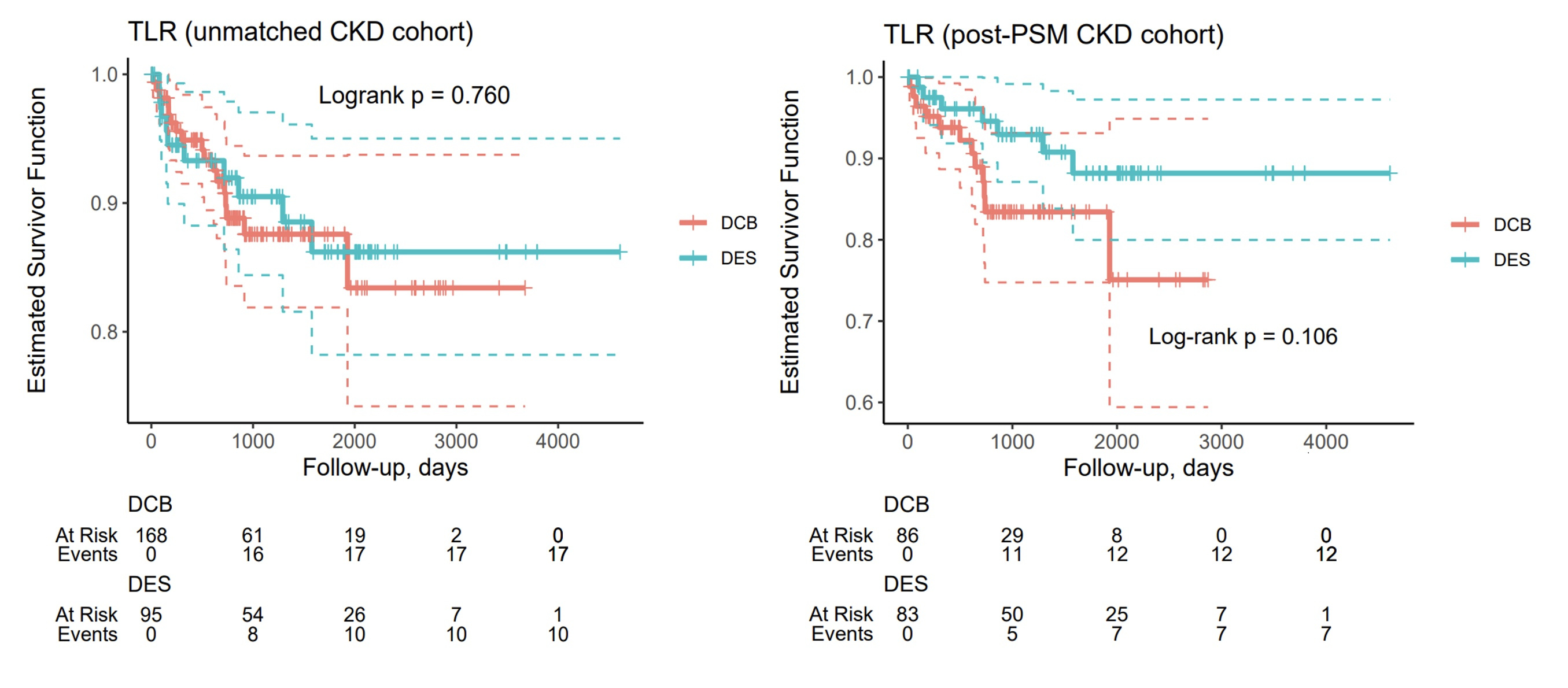

Supplement: S5 Fig — The dashed lines represent the 95% confidence intervals, while the horizontal marks indicate the patient censoring events. (JPG) [file pone.0337991.s005.jpg]

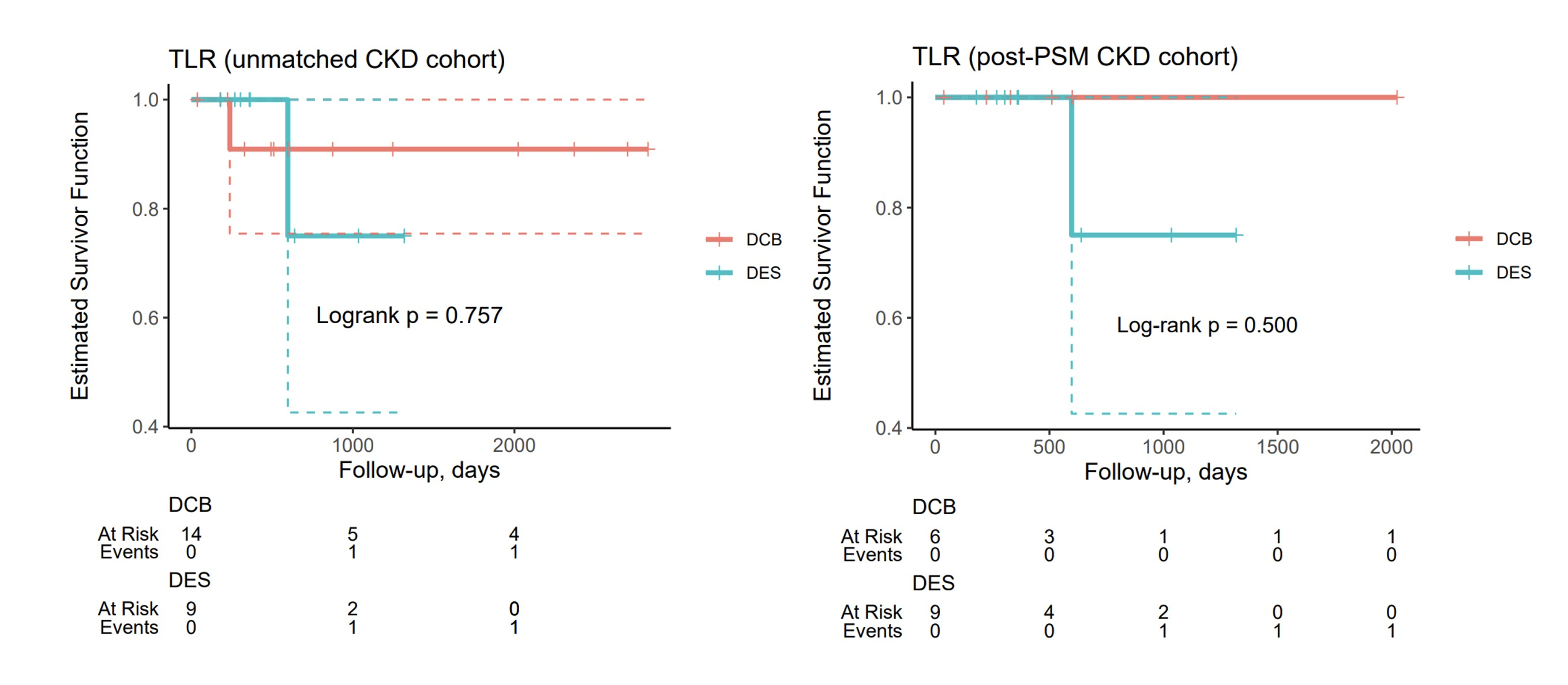

Supplement: S6 Fig — The dashed lines represent the 95% confidence intervals, while the horizontal marks indicate the patient censoring events. (JPG) [file pone.0337991.s006.jpg]
